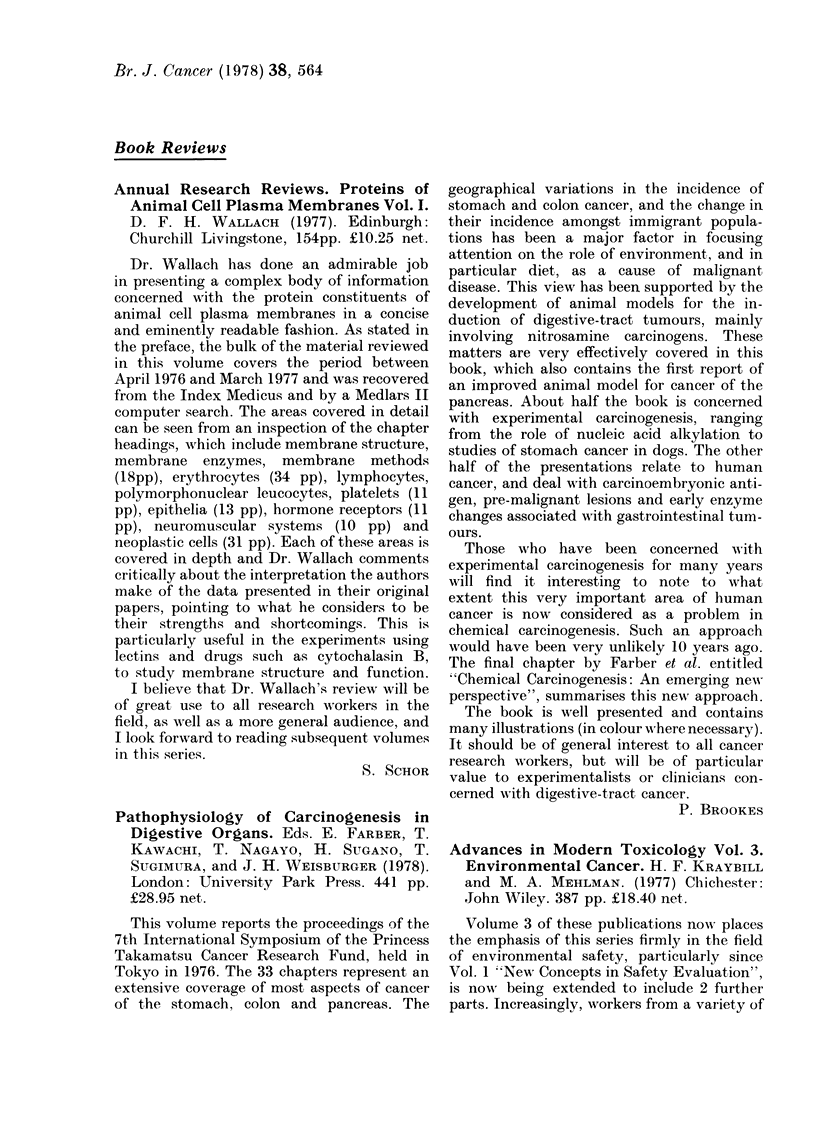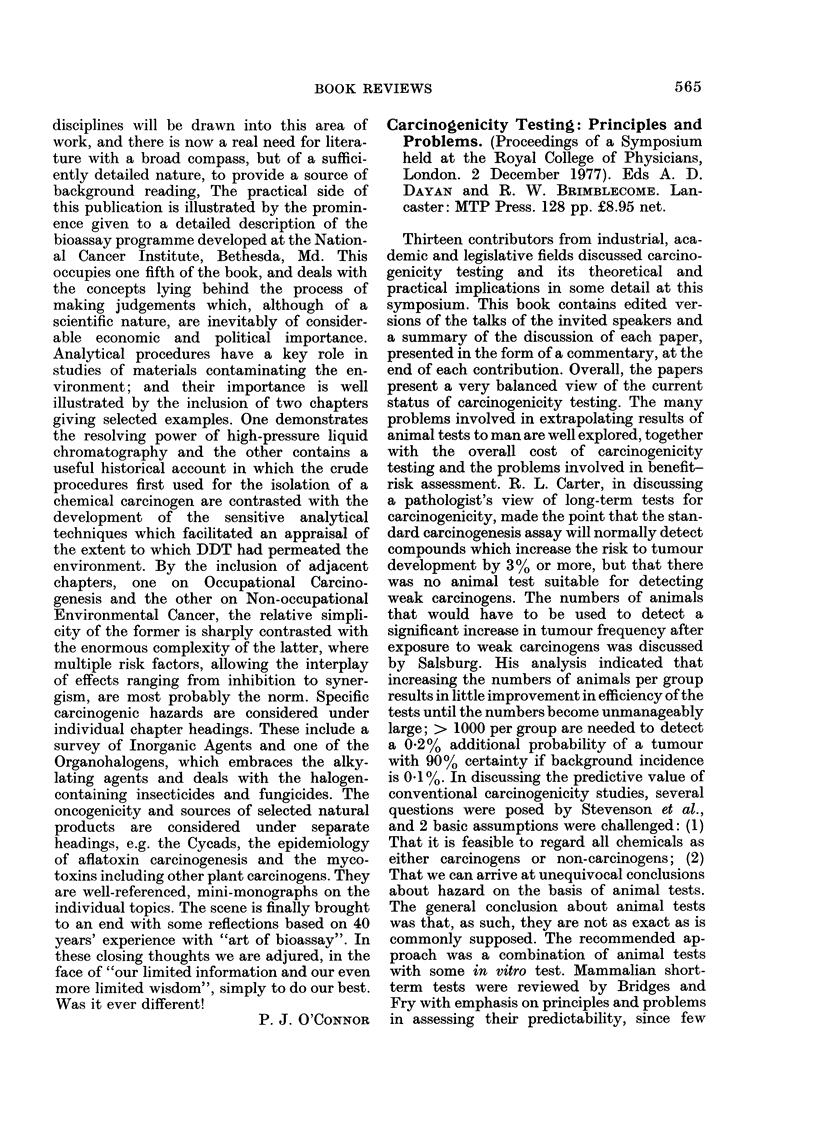# Advances in Modern Toxicology Vol. 3. Environmental Cancer

**Published:** 1978-10

**Authors:** P. J. O'Connor


**Advances in Modern Toxicology Vol. 3. Environmental Cancer.** H. F. KRAYBILL and M. A. MEHLMAN. (1977) Chichester: John Wiley. 387 pp. £18.40 net.

Volume 3 of these publications now places the emphasis of this series firmly in the field of environmental safety, particularly since Vol. 1 "New Concepts in Safety Evaluation", is now being extended to include 2 further parts. Increasingly, workers from a variety of disciplines will be drawn into this area of work, and there is now a real need for literature with a broad compass, but of a sufficiently detailed nature, to provide a source of background reading, The practical side of this publication is illustrated by the prominence given to a detailed description of the bioassay programme developed at the National Cancer Institute, Bethesda, Md. This occupies one fifth of the book, and deals with the concepts lying behind the process of making judgements which, although of a scientific nature, are inevitably of considerable economic and political importance. Analytical procedures have a key role in studies of materials contaminating the environment; and their importance is well illustrated by the inclusion of two chapters giving selected examples. One demonstrates the resolving power of high-pressure liquid chromatography and the other contains a useful historical account in which the crude procedures first used for the isolation of a chemical carcinogen are contrasted with the development of the sensitive analytical techniques which facilitated an appraisal of the extent to which DDT had permeated the environment. By the inclusion of adjacent chapters, one on Occupational Carcinogenesis and the other on Non-occupational Environmental Cancer, the relative simplicity of the former is sharply contrasted with the enormous complexity of the latter, where multiple risk factors, allowing the interplay of effects ranging from inhibition to synergism, are most probably the norm. Specific carcinogenic hazards are considered under individual chapter headings. These include a survey of Inorganic Agents and one of the Organohalogens, which embraces the alkylating agents and deals with the halogen-containing insecticides and fungicides. The oncogenicity and sources of selected natural products are considered under separate headings, e.g. the Cycads, the epidemiology of aflatoxin carcinogenesis and the mycotoxins including other plant carcinogens. They are well-referenced, mini-monographs on the individual topics. The scene is finally brought to an end with some reflections based on 40 years' experience with "art of bioassay". In these closing thoughts we are adjured, in the face of "our limited information and our even more limited wisdom", simply to do our best. Was it ever different!

P. J. O'Connor